# Antibacterial structure–activity relationship studies of several tricyclic sulfur-containing flavonoids

**DOI:** 10.3762/bjoc.12.100

**Published:** 2016-05-23

**Authors:** Lucian G Bahrin, Henning Hopf, Peter G Jones, Laura G Sarbu, Cornelia Babii, Alina C Mihai, Marius Stefan, Lucian M Birsa

**Affiliations:** 1 Department of Chemistry, “Al. I. Cuza” University of Iasi, 11 Carol I Blvd., RO-700506 Iasi, Romania; 2 Institute of Organic Chemistry, Technical University of Braunschweig, Hagenring 30, D-38106 Braunschweig, Germany; 3 Institute of Inorganic and Analytical Chemistry, Technical University of Braunschweig, Hagenring 30, D-38106 Braunschweig, Germany; 4 Department of Biology, Al. I. Cuza University of Iasi, 11 Carol I, 700506-Iasi, Romania

**Keywords:** antibacterial activity, dithiocarbamates, dithiolium salts, flavonoids, SAR studies

## Abstract

A structure–activity relationship study concerning the antibacterial properties of several halogen-substituted tricyclic sulfur-containing flavonoids has been performed. The compounds have been synthesized by cyclocondensation of the corresponding 3-dithiocarbamic flavanones under acidic conditions. The influence of different halogen substituents on the antibacterial properties has been tested against *Staphylococcus aureus* and *Escherichia coli*. Amongst the *N*,*N*-dialkylamino-substituted flavonoids, those having an *N*,*N*-diethylamino moiety exhibited good to excellent antimicrobial properties against both pathogens. Fluorine-substituted flavonoids were found to be less active than those bearing other halogen atoms.

## Introduction

The extensive use of antibiotics in human treatment and agriculture has led to the development of one of the greatest problems faced by modern medicine: multidrug-resistant bacteria. Resistance may arise via various mechanisms including changes in cell-wall permeability, target site mutation, antibiotic inactivation and the development of efflux pumps that transport the drugs out of the cell [1–2]. Clearly, the development of new types of antibiotics is strongly needed [3].

Flavonoids are a diverse class of plant secondary metabolites that share the same C_6_–C_3_–C_6_ backbone. The great structural diversity of these compounds results in a wide range of biological activities. Flavonoids can display antibacterial, antiviral, antifungal, anti-inflammatory and anticancer properties [4–10]. Their polyphenolic structure also gives them excellent anti-oxidant and cardioprotective properties [11–12].

**Figure 1:**
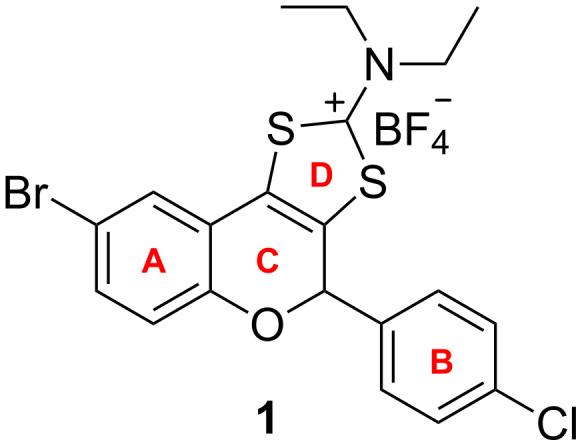
The molecular structure of tricyclic flavonoid **1**.

Our research group has recently reported the antibacterial properties of a new class of synthetic tricyclic flavonoids [13]. Given the promising results that have been obtained for compound **1** (Figure 1), we chose this compound for a structure–activity study. For this purpose we initially decided to retain the halogen substituents of the aromatic A and B rings, while changing the nature of the dialkylamino moiety at the 2 position of ring D. After the best correlation between the antibacterial activity and the nature of the dialkylamino moiety had been identified as the *N*,*N*-diethylamino group, this substituent was maintained, while the structure–activity study was extended to different halogen substituents of the A and B rings. This paper presents the results obtained, using this strategy against *Staphylococcus aureus* and *Escherichia coli*.

## Results and Discussion

### Chemistry

**Scheme 1:**
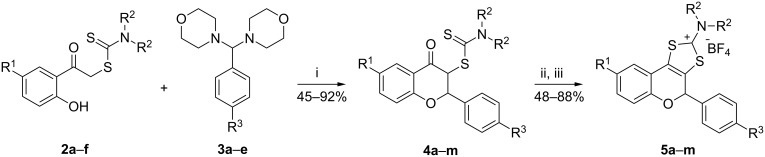
Synthesis of flavanones **4a**–**m** and tricyclic flavonoids **5a**–**m**. Conditions: i) EtOH, reflux, 4 h; ii) H_2_SO_4_/CH_3_COOH (1:3 v/v), 20 min; iii) NaBF_4_ aq.

The synthesis of the desired compounds was achieved using a method previously employed by us [14–18] through the reaction of phenacyl carbodithioates **2a**–**f** with aminals **3a**–**e** as presented in Scheme 1.

Phenacyl carbodithioates **2a**–**f**, obtained from the appropriately substituted ω-bromoacetophenones and ditihiocarbamates according to previously described procedures [19–20], and aminals **3a**–**e**, synthesized by reacting morpholine with halogenated benzaldehydes [21–22], are collected in Table 1. The individual substitution patterns and yields for flavonoids **4a**–**m** and **5a**–**m** are presented in Table 2.

**Table 1:** Phenacyl carbodithioates **2a**–**f** and halogenated aminals **3a**–**e**.

		**a**	**b**	**c**	**d**	**e**	**f**

**2**	R^1^	Br	Br	Br	Br	I	H
	NR^2^_2_	NMe_2_	pyrrolidine	piperidine	NEt_2_	NEt_2_	NEt_2_

**3**	R^3^	F	Cl	Br	I	H	–

**Table 2:** Substitution patterns and yields for flavonoids **4a**–**m** and **5a**–**m**.

Entry	**4**, **5**	R^1^	NR^2^_2_	R^3^	Yield of **4**, %	Yield of **5**, %

1	**a**	Br	NMe_2_	Cl	45	48
2	**b**	Br	pyrrolidine	Cl	92	77
3	**c**	Br	piperidine	Cl	87	70
4	**d**	Br	NEt_2_	F	85	86
5	**e**	Br	NEt_2_	Br	87	88
6	**f**	Br	NEt_2_	I	48	87
7	**g**	Br	NEt_2_	H	62	77
8	**h**	I	NEt_2_	F	70	68
9	**i**	I	NEt_2_	Cl	84	72
10	**j**	I	NEt_2_	Br	73	56
11	**k**	I	NEt_2_	I	82	62
12	**l**	I	NEt_2_	H	62	64
13	**m**	H	NEt_2_	H	85	61

**Figure 2:**
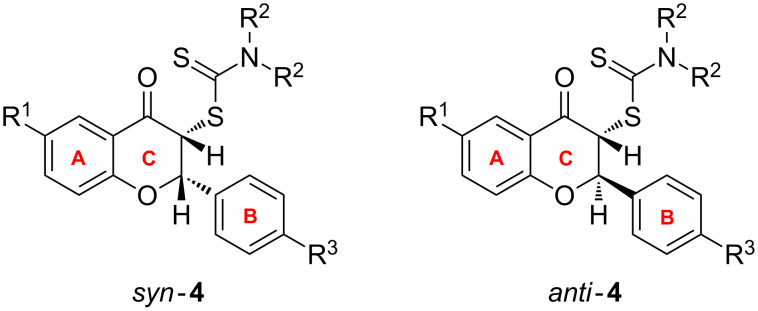
The *syn* and *anti*-isomers of flavanones **4**.

The reaction between brominated and iodinated phenacyl carbodithioates **2a**–**f** and halogenated aminals **3a–e** lead to the formation of flavanones **4a**–**m** as a mixture of diastereomers. These differ in the orientations of the H2 and H3 hydrogen atoms, which may be on the same side or on opposite sides of the ring C (Figure 2).

The outcome of the reaction was confirmed by analytical data and spectroscopical methods. The ^1^H NMR spectra of flavanones **4** all indicate the disappearance of the singlet corresponding to the methylene unit bound to the carbonyl group (~4.8 ppm). Moreover, the signal belonging to the phenolic hydrogen atom, present in the phenacyl carbodithioate ^1^H NMR spectra at ca. 12 ppm, is no longer present in the flavanone spectra. Two new sets of doublets, one from the *syn* and one from the *anti*-isomer, appear between 5 ppm and 6 ppm and correspond to the hydrogen atoms H2 and H3. These isomers can be distinguished by calculating the coupling constants, which are found to be ca. 4 Hz for the *syn* and ca. 9 Hz for the *anti*-isomers.

New signals in the aromatic region of the ^13^C NMR spectra reveal the incorporation of aromatic ring B. The EIMS spectra display the molecular ions as well as a number of characteristic fragments. For flavanones **4d**–**m** fragments formed by cleavage of the S–(CS) bond of the *N*,*N*-diethyldithiocarbamic moiety are observed. In fact, the most intense signals in the spectra belongs to the [C_5_H_10_NS]^+^ fragment at *m*/*z* = 116.1. Another common fragment at *m*/*z* = 148.0 corresponding to [C_5_H_10_NS_2_]^+^ originates from the cleavage of the C(3)–S bond in these compounds. For flavanones **4d** and **4f** crystals suitable for single crystal X-ray analysis were obtained and the results are discussed in the X-ray analysis section.

The acid-catalyzed cyclocondensation of flavanones **4a**–**m** afforded the tricyclic flavonoids **5a**–**m** in good yields [23] (Scheme 1). The formation of the 1,3-dithiolium ring is associated with characteristic spectral changes. Thus, in the ^1^H NMR spectra of flavonoids **5** the signal belonging to the hydrogen atom from the 2 position of ring C is shifted to ca. 6.85–6.95 ppm and its multiplicity changes from a doublet to a singlet. The ^13^C NMR spectra indicate the disappearance of the carbonyl and thiocarbonyl signals (ca. 191 ppm and 186 ppm) and a new signal at ca. 184 ppm appears, belonging to the positive carbon atom of the 1,3-dithiolium ring. The IR spectra of the tricyclic flavonoids **5** lack the carbonyl absorption band (ca. 1690–1680 cm^−1^) and the presence of a new broad absorption band at around 1040 cm^−1^ is attributed to the tetrafluoroborate anion. ESIMS confirms the structure of the positive molecular fragment of each tricyclic flavonoid ([M − BF_4_]^+^). Single crystals suitable for X-ray analysis were obtained for **5a** and **5b** and the results are presented in the X-ray section below.

### X-ray analyses

**Figure 3:**
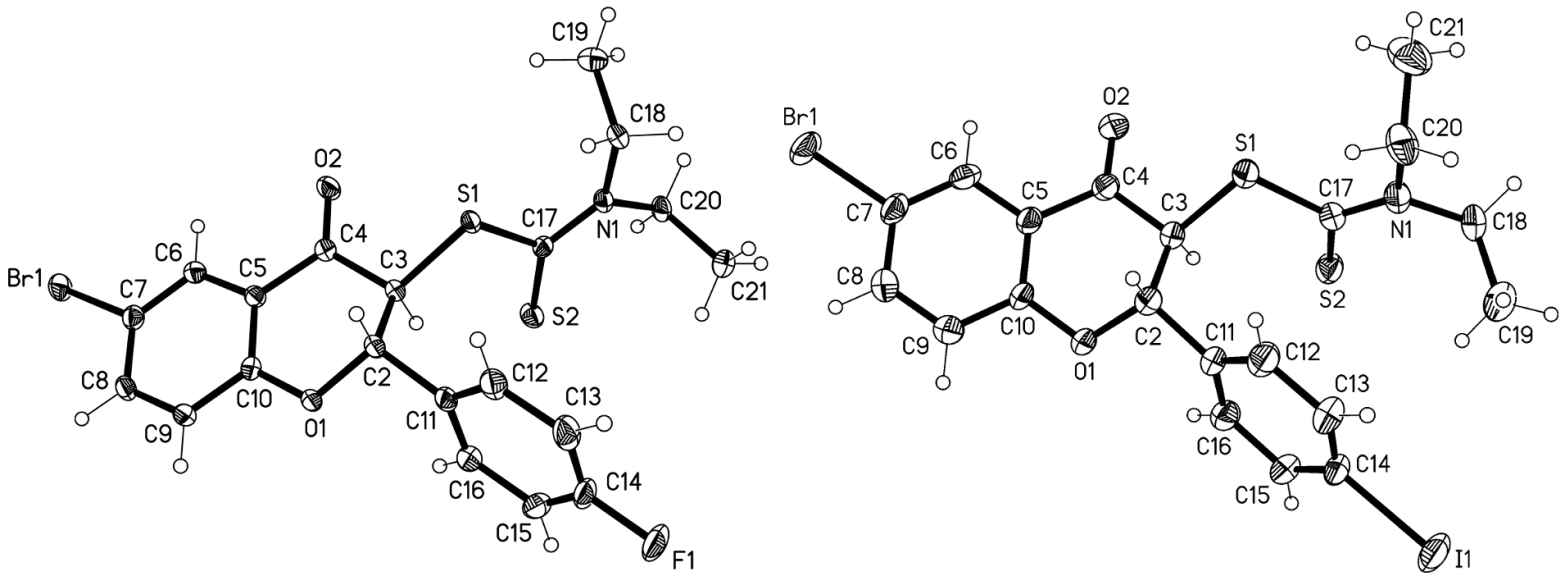
Molecular structures of **4d** (left) and **4f** (right). Ellipsoids represent 50% probability levels [24].

The crystal structures of flavanones **4d** and **4f** are presented in Figure 3.

We found that compounds of general structure **4**, despite their close chemical similarity, display conformational differences, especially regarding the ethyl groups. Relevant torsion angles for **4d** and **4f** are shown in Table 3 and are generally consistent with previously observed values (indeed, **4d** is isotypic to the compound with R^1^ = R^3^ = Cl [25]), except that **4f** shows some extreme values for ring C. Compounds **4e** and **4k** were also investigated; **4e** is isotypic to **4f** according to its cell constants (the structure was not determined), whereas **4k** proved to be seriously disordered.

**Table 3:** Selected torsion angles (°, rounded to nearest degree) for **4d** and **4f**.

	**4d**^a^	**4f**

O1–C2–C11–C16	49	41
C3–S1–C17–S2	−2	23
C17–N1–C_ethyl_–C_ethyl_	−98, −89	−91, −89
C10–O1–C2–C3	−44	−59
O1–C2–C3–C4	53	51
C2–C3–C4–C5	−35	−18
C3–C4–C5–C10	8	−8
C4–C5–C10–O1	2	3
C5–C10–O1–C2	17	32
Ring C10–O1–C2–C3–C4–C5, mean absolute torsion angle	27	29

^a^Inverted from deposited coordinates.

**Figure 4:**
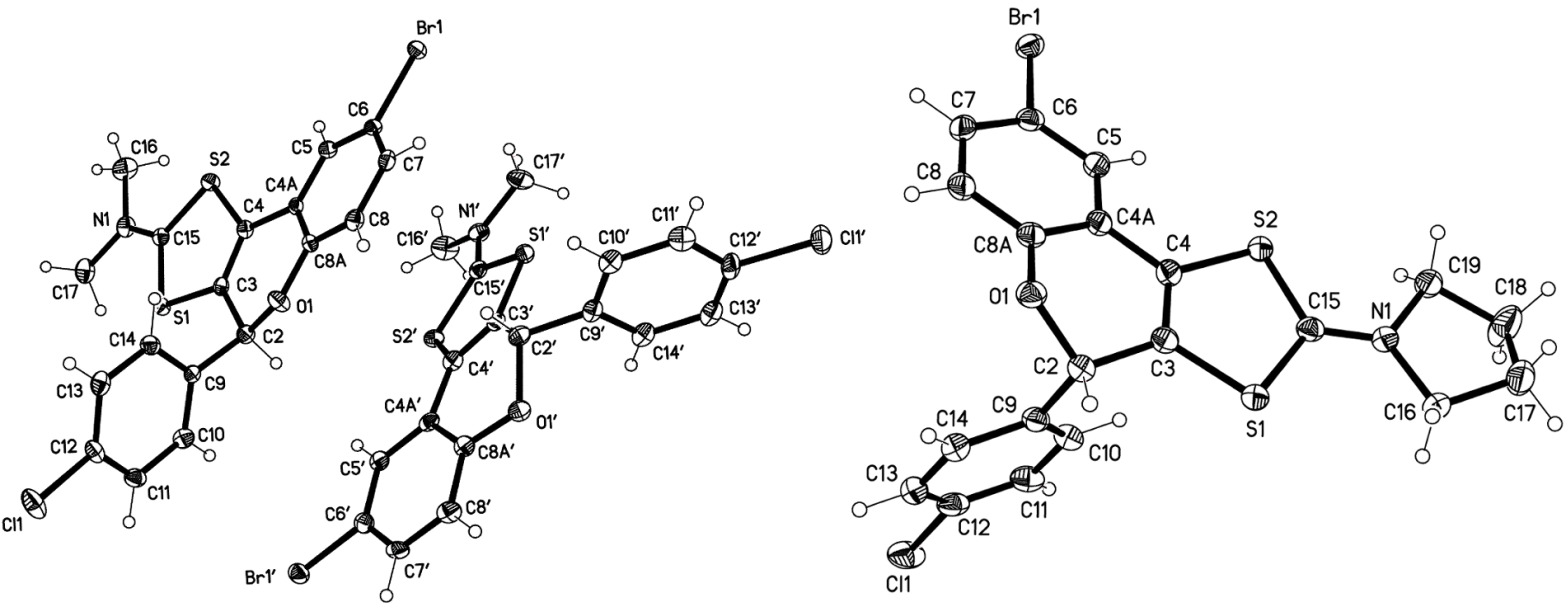
Molecular structure of **5a** (left, both independent molecules) and **5b** (right, one of two independent molecules). Ellipsoids represent 50% probability levels [26].

The crystal structures of tricyclic flavonoids **5a** and **5b** are displayed in Figure 4. Both compounds crystallize with two independent molecules in the asymmetric unit. The main possible degrees of freedom involve the ring orientations and the torsion angles of the heterocyclic ring C, although, in contrast to compounds **4**, the latter are constrained by the presence of the double bond C3=C4. Selected torsion angles are collected in Table 4, and show similar values for all four molecules, except for a flattening of the heterocyclic ring in **5a**.

**Table 4:** Selected torsion angles (°, rounded to nearest degree) for **5a** and **5b**.

	**5a**	**5b**^a^

O1–C2–C9–C10	−132, −118	−124, −131
S1–C15–N1–C17	1, 4	178, −177^b^
O1–C2–C3–C4	23, 31	36, 39
C2–C3–C4–C4A	−2, −5	−5, −7
C3–C4–C4A–C8A	−12, −13	−14, −14
C4–C4A–C8A–O1	4, 1	–1, –1
C4A–C8A–O1–C2	19, 29	34, 33
C8A–O1–C2–C3	−31, −43	−49, −51

^a^Inverted from deposited coordinates. ^b^S1–C15–N1–C19.

### In vitro antibacterial activity

The promising results obtained in our previous studies [13,27] prompted us to investigate to what degree the amino moiety and the two halogen atoms bound to tricyclic flavonoids of type **5** influence their antibacterial activity. This study also established that dithiocarbamic flavanones of type **4** display no such activity. Therefore, only flavonoids **5a**–**m** were tested against *Staphylococus aureus* (Gram positive) and *Escherichia coli* (Gram negative) in an attempt to establish an antimicrobial structure–activity relationship. Minimum inhibitory concentration (MIC) and minimum bactericidal concentration (MBC) using the standard microbroth dilution technique were recorded and the results are collected in Table 5.

**Table 5:** Minimum inhibitory concentrations and minimum bactericidal concentrations of flavonoids **5a**–**m** against *Staphylococcus aureus* and *Escherichia coli*.

Entry	Flavonoid	Bacterial strain			
		MIC (μg/mL)^a^		MBC (μg/mL)^b^	
		*S. aureus*	*E. coli*	*S. aureus*	*E. coli*

1	**5a**	7.81	15.62	–^c^	–^c^
2	**5b**	1.95	3.90	–^c^	–^c^
3	**5c**	62.5	125	–^c^	–^c^
4	**1**	0.24	3.9 [13]	0.24	7.81
5	**5d**	1.95	15.62	1.95	15.62
6	**5e**	0.48	3.9	0.97	7.81
7	**5f**	0.48	3.9	1.95	7.81
8	**5g**	1.95	7.81	1.95	250
9	**5h**	1.95	7.81	1.95	15.62
10	**5i**	0.48	3.9	1.95	15.62
11	**5j**	0.48	3.9	1.95	7.81
12	**5k**	0.48	3.9	1.95	7.81
13	**5l**	0.97	7.81	7.81	62.5
14	**5m**	62.5	62.5	250	250
15	kanamycin	1.95	7.81	1.95	7.81
16	ampicillin	7.81	7.81	–^c^	–^c^
17	control (DMSO, μL/mL)	250	125	–	–

^a^MIC = minimum inhibitory concentration. ^b^MBC = minimum bactericidal concentration. ^c^Not determined.

As mentioned above, we started the study with the synthesis and testing of tricyclic flavonoids **5a**–**d**, which differ from **1** in the amino group bound to the 1,3-dithiolium cycle (ring D). Upon testing these flavonoids against *Staphylococcus aureus* and comparing the results with those obtained for **1**, we concluded that the antibacterial activity for tricyclic flavonoids of type **5** decreases in the order NEt_2_ > pyrrolidine > NMe_2_ > piperidine (Table 5, entries 1–4). These results suggest that a larger *N*,*N*-dialkylamino group leads to a decreased antibacterial activity, with compound **5c** showing an unexpectedly high MIC value in comparison to the other three compounds of this group.

Having established this, we synthesized diethylamino-substituted flavonoids **5d**–**m**. In order to investigate the influence of halogen substituents on the biological properties of tricyclic flavonoids, we first synthesized flavonoid **5m** (R^1^ = R^3^ = H) having no halogen substituents in the structure as a reference compound. The MIC and MBC values obtained for this compound against both *S. aureus* and *E. coli* are presented in Table 5, entry 14. In the next step, we synthesized and evaluated another two tricyclic flavonoids with bromine and iodine atoms bound to ring A, **5g** (R^1^ = Br, R^3^ = H) and **5l** (R^1^ = I, R^3^ = H). With this modification, promising results concerning biological activity were obtained (Table 5, entries 8 and 13). In comparison with **5m** which showed a MIC value of 62.5 μg/mL, these compounds presented minimum inhibitory concentrations of 7.81 μg/mL against *E. coli* and 0.97–1.95 μg/mL against *S. aureus.* The bactericidal properties of flavonoids **5g** and **5l** were also promising with the exception of compound **5g** against *E. coli* for which no bactericidal effect was observed (MBC = 250 µg/mL). These results clearly indicated the importance of halogen substituents for the antibacterial activity of this class of tricyclic flavonoids. Prompted by this observation, we decided to include other halogen-substituted compounds (R^3^ = F, Cl, Br, and I) in the study.

The MIC values obtained for flavonoids **5d** (R^1^ = Br, R^3^ = F), **5e** (R^1^ = Br, R^3^ = Br), **5f** (R^1^ = Br, R^3^ = I) (Table 5, entries 5–7) and **5h** (R^1^ = I, R^3^ = F), **5i** (R^1^ = I, R^3^ = Cl), **5j** (R^1^ = I, R^3^ = Br) and **5k** (R^1^ = I, R^3^ = I) (Table 5, entries 9–12) suggested that a bulkier halogen atom in the 4-position of ring B leads to an increased bacteriostatic activity against *S. aureus* and *E. coli.* The MIC values obtained against *E. coli* were the same (3.9 µg/mL) for all tricyclic flavonoids not containing a fluorine atom in their molecular structure. This suggests that the activity against Gram negative bacteria is less dependent on the nature of the introduced heavier halogen (Cl, Br or I).

Compound **1** was found to be slightly more potent against *S. aureus* in terms of bacteriostatic properties than the other tricyclic flavonoids and showed the lowest MIC of all tested compounds against this bacterial strain. With respect to bactericidal properties, the lowest value against *S. aureus* was again recorded for flavonoid **1**. The other seven newly synthesized tricyclic flavonoids also displayed good bactericidal properties against *S. aureus*, albeit they were less potent than **1**. Moreover, the halogen atoms present in their molecular structure did not greatly influence the MBC values; six out of the seven new flavonoids leading to the same result (exception **5e**, Table 5, entry 6). This also applies to the bactericidal activities determined against *E. coli*. The tricyclic flavonoids **5e**, **5f**, **5j** and **5k** (Table 5, entries 6, 7, 11 and 12) were comparably effective as compound **1** (Table 5, entry 4). The remaining three flavonoids, of which two contain fluorine (**5d** and **5h**, Table 5, entries 5 and 9), lead to slightly higher MBC values, suggesting once again that a smaller substituent leads to a lower antibacterial activity against Gram negative microorganisms.

In order to perform a comparison with known antibiotics, both *S. aureus* and *E. coli* where exposed to ampicillin and kanamycin (Table 5, entries 15 and 16). The results indicated that the newly synthesized tricyclic flavanoids exhibit similar or even better antimicrobial properties. However, due to the lack of cytotoxicity results one may assume that the investigated structures represent a promising class of compounds.

In summary, the newly synthesized compounds exhibit good to excellent antimicrobial properties against both Gram positive and Gram negative pathogens. The introduction of halogen substituents other than fluorine in the structures of the tricyclic flavonoids leads to a significant improvement of antibacterial properties. These results suggest that the substituent size is the main factor for the change in potency rather than polarity or electronical effects. As an important influence of the *N*,*N*-dialkylamino moiety on the antibacterial activity was observed, the extension of the study on the influence of other substituents is currently on the way.

## Conclusion

A previously reported class of tricyclic flavonoids has been extended with the synthesis of thirteen new derivatives. These compounds were obtained from the corresponding 3-dithiocarbamic flavanones under acidic conditions. A study of their structure–activity relationship was performed with regard to their antibacterial properties against *Staphylococcus aureus* and *Escherichia coli*. The introduction of halogen substituents in the structures of the tricyclic flavonoids leads to a significant improvement of antibacterial properties. These results suggest that the substituent size is the main factor for the change in potency rather than polarity or electronical effects. An important dependence on the nature of the *N*,*N*-dialkylamino substituent was observed. Working with *N*,*N*-diethylamino-substituted flavonoids, good to excellent antimicrobial properties were recorded against both pathogens.

## Supporting Information

File 1 Detailed experimental procedures, supplementary spectroscopic and X-ray data.

## References

[1] Nikaido H (2009). Annu Rev Biochem.

[2] Poole K (2001). J Pharm Pharmacol.

[3] Fischbach M A, Walsh C T (2009). Science.

[4] Chukwujekwu J C, Van Heerden F R, Van Staden J (2011). Phytother Res.

[5] Martini N D, Katerere D R P, Eloff J N (2004). J Ethnopharmacol.

[6] Qian S, Fan W, Qian P, Zhang D, Wei Y, Chen H, Li X (2015). Viruses.

[7] Edziri H, Mastouri M, Mahjoub M A, Mighri Z, Mahjoub A, Verschaeve L (2012). Molecules.

[8] Angeloni C, Hrelia S (2012). Oxid Med Cell Longevity.

[9] Esmaeili M A, Farimani M M, Kiaei M (2014). Mol Cell Biochem.

[10] Shi M-D, Shiao C-K, Lee Y-C, Shih Y-W (2015). Cancer Cell Int.

[11] Wilcox L J, Borradaile N M, Huff M W (1999). Cardiovasc Drug Rev.

[12] Koga T, Meydani M (2001). Am J Clin Nutr.

[13] Bahrin L G, Apostu M O, Birsa L M, Stefan M (2014). Bioorg Med Chem Lett.

[14] Birsa M L (2002). Synth Commun.

[15] Sarbu L G, Lungu N C, Forna N C, Birsa M L (2013). Rev Chim (Bucharest, Rom).

[16] Birsa M L, Sandu I, Bahrin L G (2014). Rev Chim (Bucharest, Rom).

[17] Bahrin L G, Luca A C, Birsa M L (2014). Rev Chim (Bucharest, Rom).

[18] Bahrin L G, Asaftei I V, Sandu I G, Sarbu L G (2014). Rev Chim (Bucharest, Rom).

[19] Lungu N C, Sandu I, Chirita P, Birsa M L (2013). Rev Chim (Bucharest, Rom).

[20] Bahrin L G, Hopf H, Jones P G, Earar K, Birsa M L (2016). Rev Chim (Bucharest, Rom).

[21] Jurčík V, Wilhelm R (2004). Tetrahedron.

[22] Dezfuli M K, Saidi M R (2004). Phosphorus, Sulfur Silicon Relat Elem.

[23] Bahrin L G, Jones P G, Hopf H (2012). Beilstein J Org Chem.

[R24] 24CCDC-1452355 and -1452356 contain the supplementary crystallographic data for compounds **4d** and **4f**, respectively. These data can be obtained free of charge from The Cambridge Crystallographic Data Centre via http://www.ccdc.cam.ac.uk/data_request/cif

[R25] 25CCDC-1438848 contain the supplementary crystallographic data for a compound of type **4**, where R^1^ = R^3^ = Cl. This data can be obtained free of charge from The Cambridge Crystallographic Data Centre via http://www.ccdc.cam.ac.uk/data_request/cif

[R26] 26CCDC-1452357 and -1452358 contain the supplementary crystallographic data for compounds **5a** and **5b**, respectively. These data can be obtained free of charge from The Cambridge Crystallographic Data Centre via http://www.ccdc.cam.ac.uk/data_request/cif

[27] Babii C, Bahrin L G, Neagu A-N, Gostin I, Mihasan M, Birsa L M, Stefan M (2016). J Appl Microbiol.

