# Supplementary material for: Antibacterial structure–activity relationship studies of several tricyclic sulfur-containing flavonoids
Source: Beilstein J Org Chem. 2016 May 23;12:1065–71. doi: 10.3762/bjoc.12.100 (PMC4902053; doi:10.3762/bjoc.12.100)
Supplement: File 1 — Detailed experimental procedures, supplementary spectroscopic and X-ray data. [file Beilstein_J_Org_Chem-12-1065-s001.pdf]

Supporting information

for

Antibacterial structure–activity relationship studies of several tricyclic sulfur-containing flavonoids

Lucian G. Bahrin[1,2], Henning Hopf[2], Peter G. Jones[3], Laura G. Sarbu[1], Cornelia Babii[4], Alina C. Mihai[4], Marius Stefan*[4], and Lucian M. Birsa*[1,2]

Address: [1]Department of Chemistry, "Al. I. Cuza" University of Iasi, 11 Carol I Blvd., RO-700506 Iasi, Romania, [2]Institute of Organic Chemistry, Technical University of Braunschweig, Hagenring 30, D-38106 Braunschweig, Germany, [3]Institute of Inorganic and Analytical Chemistry, Technical University of Braunschweig, Hagenring 30, D-38106 Braunschweig, Germany and [4]Department of Biology, Al. I. Cuza University of Iasi, 11 Carol I, 700506-Iasi, Romania

Email: Lucian M. Birsa* - lbirsa@uaic.ro; Marius Stefan - stefanm@uaic.ro

*Corresponding author

Detailed experimental procedures, supplementary spectroscopic and X-ray data

Experimental

General methods: *In vitro antibacterial activity assay*

The newly synthesized target compounds were evaluated for their in vitro antimicrobial activities against *Staphylococcus aureus* ATCC 25923 as a Gram-positive bacterial strain and *Escherichia coli* ATCC 25922 as a Gram-negative bacterial strain. Microorganisms were obtained from the culture collections of the Microbiology Laboratory, Alexandru Ioan Cuza University of Iasi.

The minimum inhibitory concentration (MIC) and minimum bactericidal concentration (MBC) of these compounds were determined according to the standard microbroth dilution technique as per guidelines of the National Committee for Clinical Laboratory Standards [1]. The MIC was determined using a colorimetric microdilution technique. Briefly, testing was performed in flat-bottomed sterile 96-well microplates with lids (Becton Dickinson) in Muller–Hinton broth (MHB, Fluka). The concentration range of the test compounds was 0.122–250 μg/mL (w/v). The inoculum was prepared using an 18-hours culture adjusted by reference to the McFarland 0.5 standard (Biomerieux) and further diluted with MHB to achieve approximately 2×10^6 CFU/mL. A number of wells were reserved in each plate for control of sterility (no inoculum added), of inoculum viability (no sample added) and of the DMSO inhibitory effect. The microplates were incubated at 37 °C for 18 h. After incubation, 20 μL of resazurin solution (0.01% w/v) was added to all wells, followed by a second incubation of 1 h at 37 °C. At the end, wells were assessed visually: a color change from blue to pink or mauve was taken as indication of bacterial growth, while the highest dilution remaining blue was used to indicate the minimum inhibitory concentration (MIC). Immediately afterwards, plate counts on Muller–Hinton agar (MHA, Liofilchem) were carried out on samples from the microwells remaining blue in order to determine minimum bactericidal concentration (MBC). Three replicates of each microassay were carried out and the experiment was repeated three times. The MIC was determined as the lowest concentration at which bacteria failed to grow in MHB but were cultured when plated onto MHA. The MBC was the lowest concentration at which bacteria failed to grow in MHB and were not cultured after plating onto MHA [2].

General Remarks: Melting points: Büchi 510, uncorrected. IR: Bruker Tensor 27. ^{1}H and ^{13}C NMR: Bruker DRX 400 or DPX 300 in $CDCl_3$ or DMSO-d_6 with TMS as internal standard at rt. Chemical shifts are reported in ppm downfield from tetramethylsilane. MS: Finnigan MAT 90X, electron impact (EI, 70 eV). Electrospray ionization (ESI):

ThermoFisher Scientific LTQ-Orbitrap Velos. Typical spray voltage in positive ion mode was 2.3–2.8 kV. All reagents were commercially available and used without further purification.

Synthesis of flavanones 4; General procedure: In a similar manner as described before [3], to a solution of the appropriate carbodithioate **4** (1 mmol) in ethanol (3 mL), aminal **3** (1 mmol) was added and the resulting mixture was refluxed for 4 h. After cooling to rt, the resulting precipitate was filtered off and recrystallized from ethanol, yielding the desired compound as a crystalline solid.

6-Bromo-2-(4-chlorophenyl)-4-oxochroman-3-yl *N*,*N*-dimethyldithiocarbamate (4a): Colorless solid, yield 45% (0.20 g). M. p. 186-188 °C. ^{1}H NMR (400 MHz, $CDCl_3$): δ 3.18-3.45 (m, 3H), 3.45-3.66 (m, 3H), 3.74-3.89 (m, 2H), 6.99 (d, 1H, ^3J=8.8 Hz), 7.32-7.39 (m, 2H), 7.44-7.49 (m, 2H), 7.63 (dd, 1H, ^3J=8.8 Hz, ^4J=2.5 Hz), 8.05 (d, 1H, ^4J=2.5 Hz) ppm. ^{13}C NMR (100 MHz, $CDCl_3$): δ 41.8, 46.3, 59.9, 82.0, 114.8, 120.1, 120.3, 128.8, 129.1, 130.1, 134.8, 135.0, 139.2, 159.3, 186.4, 192.7 ppm. EI-MS (*m/z*): 88.0 ($[C_3H_6NS]^+$), 120.0 ($[C_3H_6NS_2]^+$), 197.9 ($[C_7H_3{}^{79}BrO_2]^+$), 334.9 ($[C_{15}H_9{}^{79}BrClO_2]^+$), 454.9 ($[C_{18}H_{15}{}^{79}BrClNO_2S_2]^+$). IR-ATR (cm^{-1}): $\tilde{\upsilon}$ = 2978, 2691, 1680, 1591, 1484, 1451, 1261, 974, 815, 627.

6-Bromo-2-(4-chlorophenyl)-4-oxochroman-3-yl pyrrolidine-1-carbodithioate (4b): Colorless solid, yield 92% (0.44 g). M. p. 170-172 °C. ^{1}H NMR (400 MHz, $CDCl_3$): δ 1.92-2.13 (m, 4H), 3.48-3.60 (m, 1H), 3.62-3.73 (m, 1H), 3.81-3.96 (m, 2H), 5.75-5.89 (m, 2H), 6.98 (d, 1H, ^3J=8.8 Hz), 7.35-7.38 (m, 2H), 7.45-7.50 (m, 2H), 7.63 (dd, 1H, ^3J=8.8 Hz, ^4J=2.5 Hz), 8.04 (d, 1H, ^4J=2.5 Hz) ppm. ^{13}C NMR (100 MHz, $CDCl_3$): δ 24.3, 26.1, 50.9, 55.9, 58.7, 82.1, 114.8, 120.1, 122.3, 128.8 ,129.2, 130.1, 134.8, 135.0, 139.2, 159.3, 186.5, 188.3 ppm. EI-MS (*m/z*): 114.1 ($[C_5H_8NS]^+$), 146.0 ($[C_5H_8NS_2]^+$), 197.9 ($[C_7H_3{}^{79}BrO_2]^+$), 334.9 ($[C_{15}H_9{}^{79}BrClO_2]^+$), 480.9 ($[C_{20}H_{17}{}^{79}BrClNO_2S_2]^+$). IR-ATR (cm^{-1}): $\tilde{\upsilon}$ = 2988, 2715, 1679, 1590, 1487, 1460, 1258, 983, 819, 630.

6-Bromo-2-(4-chlorophenyl)-4-oxochroman-3-yl piperidine-1-carbodithioate (4c): Colorless solid, yield 87% (0.43 g). M. p. 162-164 °C. ^{1}H NMR (400 MHz, $CDCl_3$): δ 4.02-4.40 (m, 6H), 3.72-3.96 (m, 2H), 4.08-4.35 (m, 2H), 5.79-5.90 (m, 2H), 6.99 (d, 1H, ^3J=8.8 Hz), 7.33-7.38 (m, 2H), 7.44-7.49 (m, 2H), 7.63 (dd, 1H, ^3J=8.8 Hz, ^4J=2.4 Hz), 8.04 (d,

1H, ^4J=2.4 Hz) ppm. ^{13}C NMR (100 MHz, $CDCl_3$): δ 24.1, 25.4, 26.0, 52.1, 54.3, 59.3, 82.3, 114.8, 120.1, 122.4, 128.7, 129.2, 130.1, 134.8, 134.9, 139.2, 159.3, 186.6, 191.1 ppm. EI-MS (*m/z*): 128.1 ($[C_6H_{10}NS]^+$), 160.0 ($[C_5H_{10}NS_2]^+$), 197.9 ($[C_7H_3{}^{79}BrO_2]^+$), 334.9 ($[C_{15}H_9{}^{79}BrClO_2]^+$), 494.9 ($[C_{21}H_{19}{}^{79}BrClNO_2S_2]^+$). IR-ATR ($cm^{-1}$): ῦ = 2982, 2711, 1675, 1589, 1488, 1458, 1260, 980, 817, 629.

6-Bromo-2-(4-fluorophenyl)-4-oxochroman-3-yl *N*,*N*-diethyldithiocarbamate (4d): Colorless crystals, yield 85% (0.40 g). M. p. 133-135 °C. ^{1}H-NMR (400 MHz, $CDCl_3$): δ 1.19 (t, 6H), 3.57-3.73 (m, 2H), 3.83-4.03 (m, 2H), 5.74-5.88 (m, 2H), 6.97 (d, 1H, ^3J=8.8 Hz), 6.99-7.08 (m, 2H), 7.44-7.53 (m, 2H), 7.60 (dd, 1H, ^3J=8.8 Hz, ^4J=2.5 Hz), 8.03 (d, 1H, ^3J=2.5 Hz) ppm. ^{13}C-NMR (100 MHz, $CDCl_3$): δ 10.9, 12.1, 46.8, 50.2, 59.3, 81.9, 114.2, 114.9, 119.7, 121.9, 129.2, 129.6, 131.7, 138.6, 158.9, 162.4, 186.3, 190.8 ppm. EI-MS (*m/z*): 116.1 ($[C_5H_{10}NS]^+$), 148.0 ($[C_5H_{10}NS_2]^+$), 198.0 ($[C_7H_3{}^{79}BrO_2]^+$), 319.0 ($[C_{15}H_9{}^{79}BrFO_2]^+$), 467.0 ($[C_{20}H_{19}{}^{79}BrFNO_2S_2]^+$). IR-ATR ($cm^{-1}$): ῦ = 2985, 2705, 1681, 1598, 1495, 1411, 1268, 1202, 978, 825, 520.

6-Bromo-2-(4-bromophenyl)-4-oxochroman-3-yl *N*,*N*-diethyldithiocarbamate (4e): Colorless crystals, yield 87% (0.46 g). M. p. 135-137 °C. ^{1}H-NMR (400 MHz, $CDCl_3$): δ 1.20 (t, 6H), 3.66 (q, 2H), 3.82-4.04 (m, 2H), 5.73-5.85 (m, 2H), 6.97 (d, 1H, ^3J=8.8 Hz), 7.35-7.41 (m, 2H), 7.44-7.50 (m, 2H), 7.57-7.63 (m, 1H), 8.02 (d, 1H, ^4J=2.5 Hz) ppm. ^{13}C-NMR (100 MHz, $CDCl_3$): δ 11.3, 12.5, 47.3, 50.6, 59.4, 82.3, 114.7, 120.1, 123.1, 128.7, 129.4, 130.0, 131.4, 131.5, 135.2, 139.1, 159.3, 186.5, 191.1 ppm. EI-MS (*m/z*): 116.1 ($[C_5H_{10}NS]^+$), 148.0 ($[C_5H_{10}NS_2]^+$), 198.0 ($[C_7H_3{}^{79}BrO_2]^+$), 378.9 ($[C_{15}H_9{}^{79}Br_2O_2]^+$), 526.9 ($[C_{20}H_{19}{}^{79}Br_2NO_2S_2]^+$). IR-ATR ($cm^{-1}$): ῦ = 2973, 2800, 1691, 1597, 1493, 1414, 1263, 1202, 987, 809, 500.

6-Bromo-2-(4-iodophenyl)-4-oxochroman-3-yl *N*,*N*-diethyldithiocarbamate (4f): Colorless crystals, yield 48% (0.28 g). M. p. 141-143 °C. ^{1}H-NMR (400 MHz, $CDCl_3$): δ 1.20 (t, 6H), 3.66 (q, 2H), 3.82-4.04 (m, 2H), 5.72-5.84 (m, 2H), 6.96 (d, 1H, ^3J=8.8 Hz), 7.22-7.27 (m, 2H), 7.58-7.62 (m, 1H), 7.64-7.70 (m, 2H), 8.01 (d, 1H, ^4J=2.5 Hz) ppm. ^{13}C-NMR (100 MHz, $CDCl_3$): δ 11.4, 12.5, 47.3, 50.6, 59.3, 82.4, 94.9, 114.7, 120.1, 128.9, 129.5, 130.0, 135.9, 137.4, 137.5, 139.1, 159.3, 186.5, 191.1 ppm. EI-MS (*m/z*): 116.1 ($[C_5H_{10}NS]^+$), 148.0 ($[C_5H_{10}NS_2]^+$), 198.0 ($[C_7H_3{}^{79}BrO_2]^+$), 426.9 ($[C_{15}H_9{}^{79}BrIO_2]^+$), 574.9

($[C_{20}H_{19}{}^{79}BrINO_2S_2]^+$). IR-ATR ($cm^{-1}$): $\tilde{\upsilon}$ = 2973, 2798, 1692, 1598, 1493, 1414, 1264, 1203, 986, 806, 498.

6-Bromo-2-phenyl-4-oxochroman-3-yl *N,N*-diethyldithiocarbamate (4g): Pale-yellow crystals, yield 62% (0.28 g). M. p. 144-146 °C. ^{1}H-NMR (300 MHz, $CDCl_3$): δ 1.17-1.25 (m, 6H), 3.58-3.77 (m, 2H), 3.87-4.05 (m, 2H), 5.76-5.93 (m, 2H), 7.01 (d, 1H, ^3J=8.8 Hz), 7.32-7.41 (m, 3H), 7.49-7.55 (m, 2H), 7.62 (dd, 1H, ^3J=8.8 Hz, ^4J=2.5 Hz), 8.04 (d, 1H, ^4J=2.5 Hz). ^{13}C-NMR (75 MHz, $CDCl_3$): δ 11.4, 12.5, 47.3, 50.6, 59.4, 82.9, 114.6, 120.2, 122.5, 127.6, 128.5, 129.0, 130.1, 136.3, 139.1, 159.5, 186.9, 191.4. IR-ATR (cm^{-1}): $\tilde{\upsilon}$ = 2978, 2768, 1686, 1594, 1490, 1412, 1265, 1201, 974, 824, 506.

6-Iodo-2-(4-fluorophenyl)-4-oxochroman-3-yl *N,N*-diethyldithiocarbamate (4h): Pale-yellow crystals, yield 70% (0.36 g). M. p. 142-144 °C. ^{1}H-NMR (400 MHz, $CDCl_3$): δ 1.19 (t, 6H), 3.55-3.75 (m, 2H), 3.84-4.03 (m, 2H), 5.74-5.88 (m, 2H), 6.85 (d, 1H, ^3J=8.7 Hz), 6.97-7.08 (m, 2H), 7.44-7.53 (m, 2H), 7.77 (dd, 1H, ^3J=8.7 Hz, ^4J=2.3 Hz), 8.21 (d, 1H, ^4J=2.3 Hz) ppm. ^{13}C-NMR (100 MHz, $CDCl_3$): δ 11.3, 12.5, 47.2, 50.6, 59.6, 82.3, 84.4, 115.3, 120.4, 122.8, 129.6, 132.1, 136.2, 144.7, 160.0, 162.8, 186.5, 191.2 ppm. EI-MS (*m/z*): 116.1 ($[C_5H_{10}NS]^+$), 148.0 ($[C_5H_{10}NS_2]^+$), 245.9 ($[C_7H_3IO_2]^+$), 367.0 ($[C_{15}H_9FIO_2]^+$), 515.0 ($[C_{20}H_{19}FINO_2S_2]^+$). IR-ATR ($cm^{-1}$): $\tilde{\upsilon}$ = 2981, 2706, 1680, 1591, 1494, 1423, 1269, 1201, 975, 825, 518.

6-Iodo-2-(4-chlorophenyl)-4-oxochroman-3-yl *N,N*-diethyldithiocarbamate (4i): Colorless crystals, yield 84% (0.45 g). M. p. 144-146 °C. ^{1}H-NMR (400 MHz, $CDCl_3$): δ 1.20 (t, 6H), 3.55-3.74 (m, 2H), 3.81-4.04 (m, 2H), 5.73-5.86 (m, 2H), 6.85 (d, 1H, ^3J=8.7 Hz), 7.28-7.35 (m, 2H), 7.40-7.47 (m, 2H), 7.77 (dd, 1H, ^3J=8.7 Hz, ^4J=2.3 Hz), 8.20 (d, 1H, ^4J=2.3 Hz) ppm. ^{13}C-NMR (100 MHz, $CDCl_3$): δ 11.3, 12.5, 47.2, 50.6, 59.3, 80.3, 82.2, 84.4, 120.4, 120.5, 128.4, 128.5, 129.1, 134.8, 136.2, 144.7, 160.0, 186.4, 190.7 ppm. EI-MS (*m/z*): 116.1 ($[C_5H_{10}NS]^+$), 148.0 ($[C_5H_{10}NS_2]^+$), 245.9 ($[C_7H_3IO_2]^+$), 383.0 ($[C_{15}H_9ClIO_2]^+$), 531.0 ($[C_{20}H_{19}ClINO_2S_2]^+$). IR-ATR ($cm^{-1}$): $\tilde{\upsilon}$ = 2981, 2848, 1689, 1590, 1491, 1406, 1267, 1196, 977, 825, 527.

6-Iodo-2-(4-bromophenyl)-4-oxochroman-3-yl *N,N*-diethyldithiocarbamate (4j): Pale-yellow crystals, yield 73% (0.42 g). M. p. 134-136 °C. ^{1}H-NMR (400 MHz, $CDCl_3$): δ 1.20 (t, 6H), 3.60-3.71 (m, 2H), 3.81-4.06 (m, 2H), 5.72-5.85 (m, 2H), 6.85 (d, 1H, ^3J=8.7 Hz),

7.34-7.41 (m, 2H), 7.43-7.51 (m, 2H), 7.78 (dd, 1H, ^3J=8.7 Hz, ^4J=2.3 Hz), 8.20 (d, 1H, ^4J=2.3 Hz) ppm. ^{13}C-NMR (100 MHz, $CDCl_3$): δ 11.3, 12.5, 47.7, 50.6, 59.3, 82.2, 84.4, 120.4, 123.0, 128.7, 129.4, 131.4, 131.5, 135.2, 136.2, 144.8, 159.9, 186.3, 191.1 ppm. EI-MS (*m/z*): 116.1 ($[C_5H_{10}NS]^+$), 148.0 ($[C_5H_{10}NS_2]^+$), 245.9 ($[C_7H_3IO_2]^+$), 426.9 ($[C_{15}H_9{}^{79}BrIO_2]^+$), 574.9 ($[C_{20}H_{19}{}^{79}BrINO_2S_2]^+$). IR-ATR ($cm^{-1}$): $\tilde{\upsilon}$ = 2972, 2801, 1690, 1591, 1493, 1406, 1267, 1202, 987, 809, 499.

6-Iodo-2-(4-iodophenyl)-4-oxochroman-3-yl *N,N*-diethyldithiocarbamate (4k): Pale-orange crystals, yield 82% (0.51 g). M. p. 145-147 °C. ^{1}H-NMR (400 MHz, $CDCl_3$): δ 1.20 (t, 6H), 3.66 (q, 2H), 3.81-4.06 (m, 2H), 5.71-5.83 (m, 2H), 6.85 (d, 1H, ^3J=8.7 Hz), 7.21-7.26 (m, 2H), 7.64-7.71 (m, 2H), 7.78 (dd, 1H, ^3J=8.7 Hz, ^4J=2.3 Hz), 8.20 (d, 1H, ^4J=2.3 Hz) ppm. ^{13}C-NMR (100 MHz, $CDCl_3$): δ 11.4, 12.5, 47.3, 50.6, 59.2, 82.3, 84.4, 94.9, 120.4, 122.8, 129.5, 135.9, 136.1, 137.4, 144.8, 159.9, 186.3, 191.1 ppm. EI-MS (*m/z*): 116.1 ($[C_5H_{10}NS]^+$), 148.0 ($[C_5H_{10}NS_2]^+$), 245.9 ($[C_7H_3IO_2]^+$), 474.9 ($[C_{15}H_9I_2O_2]^+$), 622.9 ($[C_{20}H_{19}I_2NO_2S_2]^+$). IR-ATR ($cm^{-1}$): $\tilde{\upsilon}$ = 2973, 2803, 1690, 1591, 1493, 1403, 1266, 1202, 986, 805, 497.

6-Iodo-2-phenyl-4-oxochroman-3-yl *N,N*-diethyldithiocarbamate (4l): Pale-yellow crystals, yield 62% (0.31 g). M. p. 145-147 °C. ^{1}H-NMR (300 MHz, $CDCl_3$): δ 1.16-1.28 (m, 6H), 3.59-3.77 (m, 2H), 3.88-4.06 (m, 2H), 5.79-5.92 (m, 2H), 6.88 (d, 1H, ^3J=8.7 Hz), 7.31-7.41 (m, 3H), 7.49-7.55 (m, 2H), 7.80 (dd, 1H, ^3J=8.7 Hz, ^4J=2.3 Hz), 8.23 (d, 1H, ^4J=2.3 Hz). ^{13}C-NMR (75 MHz, $CDCl_3$): δ 11.4, 12.5, 47.3, 50.6, 59.3, 82.8, 84.2, 120.5, 123.0, 127.6, 128.5, 129.0, 136.1, 136.2, 144.7, 160.2, 186.7, 191.5. IR-ATR (cm^{-1}): $\tilde{\upsilon}$ = 2977, 2710, 1681, 1592, 1492, 1407, 1268, 1201, 974, 825, 650.

2-Phenyl-4-oxochroman-3-yl *N,N*-diethyldithiocarbamate (4m): Colorless crystals, yield 85% (0.31 g). M. p. 165-167 °C. ^{1}H-NMR (300 MHz, $CDCl_3$): δ 1.16-1.26 (m, 6H), 3.58-3.78 (m, 2H), 3.88-4.05 (m, 2H), 5.80-5.95 (m, 2H), 7.06-7.14 (m, 2H), 7.31-7.42 (m, 3H), 7.51-7.60 (m, 3H), 7.92-7.98 (m, 1H). ^{13}C-NMR (75 MHz, $CDCl_3$): δ 11.4, 12.5, 47.2, 50.5, 59.9, 82.8, 118.1, 121.2, 121.8, 127.72, 127.73, 128.4, 128.8, 136.5, 136.7, 160.7, 187.9, 191.9. IR-ATR (cm^{-1}): $\tilde{\upsilon}$ = 2980, 2764, 1687, 1602, 1493, 1419, 1297, 1198, 975, 755, 630.

Synthesis of tricyclic flavonoids 5; General procedure: To a mixture of sulfuric acid (0.25 mL) and acetic acid (0.75 mL), the appropriate flavanone **4** (0.3 mmol) was added and the resulting solution was heated to 80 °C for 20 min. The reaction mixture was then left to cool to room temperature and a solution of sodium tetrafluoroborate (110 mg) in water (5 mL) was added dropwise, with vigorous stirring. The resulting precipitate was then filtered off, washed thoroughly with water and recrystallized from ethanol, yielding the desired product in the form of colorless crystals.

2-*N*,*N*-Dimethylamino-8-bromo-4-(4-chlorophenyl)-4*H*-1,3-dithiol[4,5-*c*]chromen-2-ylium tetrafluoroborate (5a): Pale-yellow crystals, yield 48% (0.08 g). M. p. >220 °C. ^{1}H NMR (400 MHz, DMSO-d_6): δ 3.54 (s, 3H), 3.60 (s, 3H), 6.86 (s, 1H), 7.04 (d, 1H, ^{3}J=8.7 Hz), 7.49-7.57 (m, 5H), 7.74 (d, 1H, ^{4}J=2.3 Hz) ppm. ^{13}C NMR (100 MHz, DMSO-d_6): δ 48.1, 48.4, 75.0, 114.8, 118.7, 119.9, 127.6, 127.8, 129.2, 129.7, 129.9, 135.0, 135.2, 136.0, 150.4, 185.5 ppm. ESI-MS (*m/z*, $[C_{18}H_{14}{}^{79}BrClNOS_2]^+$): 437.9393 . IR-ATR (cm^{-1}): $\tilde{\upsilon}$ = 1596, 1531, 1454, 1262, 1211, 1044, 847, 525.

2-(Pyrrolidin-1-yl)-8-bromo-4-(4-chlorophenyl)-4*H*-1,3-dithiol[4,5-*c*]chromen-2-ylium tetrafluoroborate (5b): Pale-yellow crystals, yield 77% (0.13 g). M. p. >220 °C. ^{1}H NMR (400 MHz, DMSO-d_6): δ 2.37-2.48 (m, 4H), 3.92-3.98 (m, 2H), 6.77-6.81 (m, 2H), 6.79 (s, 1H), 7.04 (d, 1H, ^{3}J=8.7 Hz), 7.52-7.59 (m, 3H), 7.63-7.67 (m, 3H) ppm. ^{13}C NMR (100 MHz, DMSO-d_6): δ 26.4, 26.5, 57.9, 58.2, 75.7, 114.6, 118.5, 119.4, 127.1, 127.9, 129.3, 129.4, 129.5, 134.7, 135.6, 135.8, 150.7, 180.1 ppm. ESI-MS (*m/z*, $[C_{20}H_{16}{}^{79}BrClNOS_2]^+$): 463.9554 . IR-ATR (cm^{-1}): $\tilde{\upsilon}$ = 1592, 1540, 1465, 1269, 1201, 1051, 837, 514.

2-(Piperidin-1-yl)-8-bromo-4-(4-chlorophenyl)-4*H*-1,3-dithiol[4,5-*c*]chromen-2-ylium tetrafluoroborate (5c): Colorless solid, yield 70% (0.12 g). M. p. 150 °C (dec). ^{1}H NMR (400 MHz, DMSO-d_6): δ 1.64-1.72 (m, 2H), 1.77-1.84 (m, 2H), 1.84-1.91 (m, 2H), 3.80-3.85 (m, 2H), 3.87-3.93 (m, 2H), 6.83 (s, 1H), 7.04 (d, 1H, ^{3}J=8.7 Hz), 7.51-7.57 (m, 5H), 7.66 (d, 1H, ^{4}J=2.3 Hz) ppm. ^{13}C NMR (100 MHz, DMSO-d_6): δ 21.5, 24.9, 57.7, 58.1, 75.1, 114.8, 118.6, 119.9, 126.5, 127.4, 128.2, 129.7, 129.9, 134.9, 135.2, 136.0, 150.5, 184.4 ppm. ESI-MS (*m/z*, $[C_{21}H_{18}{}^{79}BrClNOS_2]^+$): 477.9710 . IR-ATR (cm^{-1}): $\tilde{\upsilon}$ = 1594, 1538, 1460, 1259, 1215, 1040, 828, 517.

2-*N*,*N*-Diethylamino-8-bromo-4-(4-fluorophenyl)-4*H*-1,3-dithiol[4,5-*c*]chromen-2-ylium tetrafluoroborate (5d): Colorless crystals, yield 86% (0.14 g). M. p. >220 °C. ^{1}H-NMR (400 MHz, DMSO-d_6): δ 1.32 (t, 3H), 1.41 (t, 3H), 3.79-4.01 (m, 4H), 6.83 (s, 1H), 7.03 (d, 1H, ^{3}J=8.7 Hz), 7.26-7.35 (m, 2H), 7.54 (dd, 1H, ^{3}J=8.7 Hz, ^{4}J=2.3 Hz), 2.53-2.61 (m, 2H), 7.74 (d, 1H, ^{4}J=2.3 Hz) ppm. ^{13}C-NMR (100 MHz, DMSO-d_6): δ 10.0, 10.3, 54.1, 54.3, 74.6, 114.2, 116.1, 118.2, 119.4, 126.8, 127.0, 128.5, 130.1, 132.8, 134.4, 149.9, 162.8, 184.3 ppm. ESI-MS (*m/z*, $[C_{20}H_{18}{}^{79}BrFNOS_2]^+$): 450.0001. IR-ATR (cm^{-1}): ῦ = 1598, 1538, 1469, 1221, 1046, 1033, 817, 503.

2-*N*,*N*-Diethylamino-8-bromo-4-(4-bromophenyl)-4*H*-1,3-dithiol[4,5-*c*]chromen-2-ylium tetrafluoroborate (5e): Colorless crystals, yield 88% (0.16 g). M. p. 209-211 °C. ^{1}H-NMR (400 MHz, DMSO-d_6): δ 1.32 (t, 3H), 1.40 (t, 3H), 3.81-4.00 (m, 4H), 6.83 (s, 1H), 7.04 (d, 1H, ^{3}J=8.7 Hz), 7.42-7.49 (m, 2H), 7.54 (dd, 1H, ^{3}J=8.7 Hz, ^{4}J=2.3 Hz), 7.64-7.71 (m, 2H), 7.74 (d, 1H, ^{4}J=2.3 Hz) ppm. ^{13}C-NMR (100 MHz, DMSO-d_6): δ 10.1, 10.3, 54.1, 54.3, 74.6, 114.3, 118.2, 119.4, 123.4, 126.9, 127.1, 128.1, 129.7, 132.1, 134.4, 135.8, 149.9, 184.4 ppm. ESI-MS (*m/z*, $[C_{20}H_{18}{}^{79}Br_2NOS_2]^+$): 509.9194. IR-ATR (cm^{-1}): ῦ = 1593, 1536, 1468, 1222, 1046, 1033, 830, 518.

2-*N*,*N*-Diethylamino-8-bromo-4-(4-iodophenyl)-4*H*-1,3-dithiol[4,5-*c*]chromen-2-ylium tetrafluoroborate (5f): Colorless crystals, yield 87% (0.17 g). M. p. >220 °C. ^{1}H-NMR (400 MHz, DMSO-d_6): δ 1.32 (t, 3H), 1.40 (t, 3H), 3.79-3.99 (m, 4H), 6.80 (s, 1H), 7.03 (d, 1H, ^{3}J=8.7 Hz), 7.27-7.32 (m, 2H), 7.54 (dd, 1H, ^{3}J=8.7 Hz, ^{4}J=2.3 Hz), 7.74 (d, 1H, ^{4}J=2.3 Hz), 7.80-7.86 (m, 2H) ppm. ^{13}C-NMR (100 MHz, DMSO-d_6): δ 10.1, 10.3, 54.1, 54.3, 74.8, 97.0, 114.3, 118.2, 119.4, 126.8, 127.0, 128.1, 129.6, 134.4, 136.2, 137.9, 149.9, 184.3 ppm. ESI-MS (*m/z*, $[C_{20}H_{18}{}^{79}BrINOS_2]^+$): 557.9057. IR-ATR (cm^{-1}): ῦ = 1592, 1537, 1469, 1221, 1048, 1031, 802, 518.

2-*N*,*N*-Diethylamino-8-bromo-4-phenyl-4*H*-1,3-dithiol[4,5-*c*]chromen-2-ylium tetrafluoroborate (5g): Colorless crystals, yield 77% (0.12 g). M. p. >220 °C. ^{1}H-NMR (300 MHz, DMSO-d6): δ 1.31 (t, 3H), 1.40 (t, 3H), 3.80-3.99 (m, 4H), 6.82 (s, 1H), 7.04 (d, 1H, ^{3}J=8.7 Hz), 7.44-7.57 (m, 6H), 7.72-7.77 (m, 1H). ^{13}C-NMR (75 MHz, DMSO-d6): δ 10.6, 10.8, 54.6, 54.8, 75.9, 114.7, 118.8, 119.9, 127.2, 127.5, 128.0, 129.2, 129.6, 130.5, 134.9, 137.0, 150.7, 184.8. IR-ATR (cm^{-1}): ῦ = 1597, 1536, 1467, 1221, 1045, 991, 772, 714.

2-*N*,*N*-Diethylamino-8-iodo-4-(4-fluorophenyl)-4*H*-1,3-dithiol[4,5-*c*]chromen-2-ylium tetrafluoroborate (5h): Colorless crystals, yield 68% (0.12 g). M. p. >220 °C. ^{1}H-NMR (400 MHz, DMSO-d_6): δ 1.32 (t, 3H), 1.40 (t, 3H), 3.79-4.00 (m, 4H), 6.82 (s, 1H), 6.88 (d, 1H, ^{3}J=8.5 Hz), 7.25-7.35 (m, 2H), 7.52-7.60 (m, 2H), 7.68 (dd, 1H, ^{3}J=8.5 Hz, ^{4}J=2.0 Hz), 7.81 (d, 1H, ^{4}J=2.0 Hz) ppm. ^{13}C-NMR (100 MHz, DMSO-d_6): δ 10.1, 10.3, 54.1, 54.2, 74.5, 85.9, 116.0, 118.5, 119.6, 126.7, 128.1, 130.1, 132.5, 132.9, 140.3, 150.5, 162.8, 184.3 ppm. ESI-MS (*m/z*, $[C_{20}H_{18}FINOS_2]^+$): 497.9843. IR-ATR (cm^{-1}): $\tilde{\upsilon}$ = 1594, 1536, 1468, 1218, 1031, 816, 533.

2-*N*,*N*-Diethylamino-8-iodo-4-(4-chlorophenyl)-4*H*-1,3-dithiol[4,5-*c*]chromen-2-ylium tetrafluoroborate (5i): Colorless crystals, yield 72% (0.13 g). M. p. >220 °C. ^{1}H-NMR (400 MHz, DMSO-d_6): δ 1.32 (t, 3H), 1.40 (t, 3H), 3.79-4.00 (m, 4H), 6.83 (s, 1H), 6.89 (d, 1H, ^{3}J=8.5 Hz), 7.49-7.56 (m, 4H), 7.68 (dd, 1H, ^{3}J=8.5 Hz, ^{4}J=2.0 Hz), 7.81 (d, 1H, ^{4}J=2.0 Hz) ppm. ^{13}C-NMR (100 MHz, DMSO-d_6): δ 10.1, 10.3, 54.1, 54.2, 74.4, 86.0, 118.5, 119.6, 126.8, 127.8, 129.1, 129.5, 132.5, 134.6, 135.5, 140.3, 150.4, 184.4 ppm. ESI-MS (*m/z*, $[C_{20}H_{18}ClINOS_2]^+$): 513.9557. IR-ATR (cm^{-1}): $\tilde{\upsilon}$ = 1591, 1532, 1466, 1222, 1045, 1034, 803, 519.

2-*N*,*N*-Diethylamino-8-iodo-4-(4-bromophenyl)-4*H*-1,3-dithiol[4,5-*c*]chromen-2-ylium tetrafluoroborate (5j): Colorless crystals, yield 56% (0.11 g). M. p. >220 °C. ^{1}H-NMR (400 MHz, DMSO-d_6): δ 1.32 (t, 3H), 1.40 (t, 3H), 3.79-3.99 (m, 4H), 6.82 (s, 1H), 6.89 (d, 1H, ^{3}J=8.5 Hz), 7.42-7.48 (m, 2H), 7.64-7.71 (m, 3H), 7.81 (d, 1H, ^{4}J=2.0 Hz) ppm. ^{13}C-NMR (100 MHz, DMSO-d_6): δ 10.1, 10.3, 54.1, 54.2, 74.5, 86.0, 118.5, 119.6, 123.4, 126.8, 127.7, 129.7, 132.1, 132.5, 135.9, 140.3, 150.4, 184.4 ppm. ESI-MS (*m/z*, $[C_{20}H_{18}{}^{79}BrINOS_2]^+$): 557.9057. IR-ATR (cm^{-1}): $\tilde{\upsilon}$ = 1591, 1535, 1467, 1222, 1050, 841, 522.

2-*N*,*N*-Diethylamino-8-iodo-4-(4-iodophenyl)-4*H*-1,3-dithiol[4,5-*c*]chromen-2-ylium tetrafluoroborate (5k): Colorless crystals, yield 62% (0.13 g). M. p. >220 °C. ^{1}H-NMR (400 MHz, DMSO-d_6): δ 1.32 (t, 3H), 1.40 (t, 3H), 3.79-3.99 (m, 4H), 6.79 (s, 1H), 6.88 (d, 1H, ^{3}J=8.5 Hz), 7.26-7.32 (m, 2H), 7.68 (dd, 1H, ^{3}J=8.5 Hz, ^{4}J=2.0 Hz), 7.80 (d, 1H, ^{4}J=2.0 Hz), 7.81-7.87 (m, 2H) ppm. ^{13}C-NMR (100 MHz, DMSO-d_6): δ 10.1, 10.3, 54.1, 54.2, 74.7, 86.0, 97.0, 118.5, 119.6, 126.7, 127.7, 129.6, 132.5, 136.2, 137.9, 140.3, 150.4, 184.3

ppm. ESI-MS (*m/z*, $[C_{20}H_{18}I_2NOS_2]^+$): 605.8916. IR-ATR (cm^{-1}): $\tilde{\upsilon}$ = 1593, 1534, 1467, 1223, 1053, 1034, 841, 520.

2-*N*,*N*-Diethylamino-8-iodo-4-phenyl-4*H*-1,3-dithiol[4,5-*c*]chromen-2-ylium tetrafluoroborate (5l): Colorless crystals, yield 77% (0.11 g). M. p. >220 °C. ^{1}H-NMR (300 MHz, DMSO-d6): δ 1.31 (t, 3H), 1.40 (t, 3H), 3.81-3.98 (m, 4H), 6.81 (s, 1H), 6.89 (d, 1H, ^3J=8.5 Hz), 7.45-7.52 (m, 5H), 7.68 (dd, 1H, ^3J=8.5 Hz, ^4J=2.0 Hz), 7.81 (d, 1H, ^4J=2.0 Hz). ^{13}C-NMR (75 MHz, DMSO-d6): δ 10.6, 10.8, 54.6, 54.7, 75.8, 86.3, 119.1, 120.1, 127.1, 128.0, 128.8, 129.6, 130.5, 133.0, 137.1, 140.8, 151.2, 184.8. IR-ATR (cm^{-1}): $\tilde{\upsilon}$ = 1593, 1535, 1467, 1222, 1044, 1031, 829, 700.

2-*N*,*N*-Diethylamino-4-phenyl-4*H*-1,3-dithiol[4,5-*c*]chromen-2-ylium tetrafluoroborate (5m): Colorless crystals, yield 77% (0.08 g). M. p. >220 °C. ^{1}H-NMR (300 MHz, DMSO-d6): δ 1.31 (t, 3H), 1.40 (t, 3H), 3.80-4.00 (m, 4H), 6.78 (s, 1H), 7.05-7.09 (m, 1H), 7.11-7.16 (m, 1H), 7.36-7.41 (m, 1H), 7.44-7.49 (m, 4H), 7.50-7.55 (m, 2H). ^{13}C-NMR (75 MHz, DMSO-d6): δ 10.7, 10.8, 54.5, 54.7, 75.7, 116.8, 117.7, 123.6, 125.3, 127.8, 128.0, 128.4, 129.6, 130.4, 132.6, 137.4, 151.5, 184.9. IR-ATR (cm^{-1}): $\tilde{\upsilon}$ = 1603, 1539, 1448, 1199, 1045, 1034, 761, 519.

X-Ray structure determination

Crystal data are summarized in Table S1. Crystals were mounted in inert oil on glass fibres and transferred to the cold gas stream of an Oxford Diffraction Xcalibur A or Nova E diffractometer. Intensity measurements were performed using monochromated MoKα radiation (**5a**) or mirror-focussed CuKα radiation (other structures), respectively. Absorption corrections were based on multi-scans. Structures were refined anisotropically on F^2 using the program SHELXL-97 [4]. Hydrogen atoms were included using a riding model or rigid methyl groups. *Exceptions and special features*: For **5a**, the absorption correction was based on indexed faces. The Flack parameter refined to −0.003(4). The

methyl group at C16 was modeled using a hexagon of half-occupied hydrogen sites (AFIX 127). For **5b**, the structure consists of two formula units related by a pseudotranslation of *a*/2 [5]. There are small differences between the two independent cations, as shown e.g. by the torsion angles C3–C2–C9–C10 (1.8 and 10.5°, indicating a slightly different orientation of the chlorophenyl rings). Both BF_4 anions are disordered; despite the use of appropriate restraints, some of the bond lengths and angles are unsatisfactory. Dimensions of disordered groups should always be interpreted with caution.

Table S1: Crystallographic data for **4d**, **4f**, **5a** and **5b**

Compound	**4d**	**4f**	**5a**	**5b**
Formula	$C_{20}H_{19}BrFNO_2S_2$	$C_{20}H_{19}BrINO_2S_2$	$C_{18}H_{14}BBrClF_4NOS_2$	$C_{20}H_{16}BBrClF_4NOS_2$
M_r	468.39	576.29	526.59	552.63
Temperature (K)	100	100	100	100
Crystal habit	colourless tablet	colourless tablet	colourless tablet	colourless plate
Crystal size (mm)	0.16 × 0.12 × 0.04	0.15 × 0.10 × 0.05	0.4 × 0.3 × 0.1	0.15 × 0.10 × 0.03
Crystal system	triclinic	monoclinic	monoclinic	monoclinic
Space group	*P*(-1)	$P2_1/c$	*Cc*	*P*(-1)
Cell dimensions:				
a (Å)	9.6732(7)	6.8040(4)	18.0870(5)	12.4016(5)
b (Å)	10.1196(10)	28.858(2)	9.4058(3)	13.6122(5)
c (Å)	11.7093(8)	10.9343(8)	23.8085(7)	15.5358(6)
α (°)	69.841(8)	90	90	65.119(4)
β (°)	84.877(6)	98.114(6)	103.092(3)	67.336(4)
γ (°)	67.504(8)	90	90	66.515(4)
Cell volume (Å^3)	992.91	2125.5	3945.1	2106.15
Z	2	4	8	4
D_x (g cm^{-3})	1.567	1.801	1.773	1.743
Radiation, wavelength (Å)	Cu $K\alpha$, 1.54184 Å	Cu $K\alpha$, 1.54184 Å	Mo $K\alpha$, 0.71073 Å	Cu $K\alpha$, 1.54184 Å
μ (mm^{-1})	5.0	16.0	2.5	6.1
2θ(max) (°)	151.3	151.0	60	152.6
Reflections collected	36124	34420	50593	88007
Independent reflections	4123	4401	11208	8795
R(int)	0.042	0.066	0.049	0.055
Transmissions	0.624 – 1.000	0.391 – 1.000	0.983 – 0.995	0.568 – 1.000

Data/parameters	4123/247	4401/246	11208/527	8795/593
Goodness-of-fit on F^2	1.04	1.06	1.05	1.02
$wR2$ (all reflections)	0.063	0.102	0.067	0.079
$R1$ ($F > 4\sigma(F)$)	0.025	0.043	0.035	0.029
Max. $\Delta\rho$ (e Å^{-3})	0.36	2.1	0.63	0.54

References

1. National Committee for Clinical Laboratory Standards, 2014. Performance standards for antimicrobial susceptibility testing - Methods for dilution antimicrobial susceptibility tests for bacteria that grow aerobically. Approved Standard M07-A9. NCCLS, Mayne, Pa.

2. Burt, A.S.; Reinders, R.D. *Lett. Applied Microbiol.*, **2003**, *36*, 162-167.

3. Bahrin, L. G.; Jones, P. G.; Hopf, H. *Beilstein J. Org. Chem*. **2012**, *8*, 1999–2003.

4. Sheldrick, G. M., *Acta Cryst*. **2008**, *A64*, 112-122.

5. The original cell as determined automatically was triclinic with cell constants *a* = 6.2011, *b* = 12.5083, *c* = 14.3376 Å, α = 71.58, β = 89.14, γ = 86.44°. Inspection of the diffraction pattern showed additional weak but significant reflections corresponding to a doubling of the *a*-axis. The smaller cell can be transformed to the larger cell with the matrix / 2 0 0 / 1 −1 0 / 1 0 −1 / and the inverse matrix for the reverse transformation is / 0.5 0 0 / 0.5 −1 0 / 0.5 0 −1 /.
